# Enhancing Electromigration Lifetime Through Controlled Reduction of Bismuth Content in Sn-Bi-Ag Solder Interconnects

**DOI:** 10.3390/ma18245672

**Published:** 2025-12-17

**Authors:** Shengbo Wang, Shuai Meng, Houlin Liu, Mingliang Huang

**Affiliations:** Electronic Packaging Materials Laboratory, School of Materials Science and Engineering, Dalian University of Technology, Dalian 116024, China

**Keywords:** electromigration, reliability, Sn-Bi-Ag solder, Bi content, atomic diffusion flux, lifetime

## Abstract

This study systematically investigates the influence of Bi content on the electromigration (EM) lifetime of low-temperature Cu/Sn-xBi-1Ag (600 μm)/Cu interconnects, where x = 57, 47 and 40 wt.%. The intrinsically higher product of diffusivity and effective charge number (DZ*) for Bi compared to Sn drives pronounced preferential migration of Bi atoms towards the anode, resulting in progressive β-Sn/Bi phase separation and linear thickening of a Bi-rich layer at the anode. Reducing the Bi content suppresses the EM-induced atomic flux (*J_EM_*) through three principal mechanisms: (i) a decrease in the atomic concentration of mobile Bi atoms; (ii) a reduction in electrical resistivity that weakens the electron wind force; and (iii) an increase in lattice diffusion distance that lowers the effective diffusion coefficient (*D_eff_*). The suppression of *J_EM_* directly governs the thickening kinetics of anodic Bi layer, as evidenced by the close agreement between the calculated (1:0.40:0.23) and measured (1:0.45:0.26) anodic Bi layer growth rate ratios. Consequently, the EM lifetime is significantly extended from 62.3 h (Sn-57Bi-1Ag) to 164.9 h (Sn-47Bi-1Ag) and 414.1 h (Sn-40Bi-1Ag), representing 2.6-fold and 6.6-fold improvements, respectively. This study highlights that reducing the Bi content is an effective strategy for enhancing the EM reliability of Sn-Bi-Ag solder interconnects.

## 1. Introduction

The relentless pursuit of miniaturization in advanced electronic packaging is driving the scaling of solder bump diameters from hundreds of microns towards the sub-10-micron regime. This geometric downsizing leads to a quadratic increase in current density through each solder bump as its diameter is reduced by half, reaching magnitudes on the order of 10^4^ A/cm^2^. Such high current densities generate significant Joule heating, which substantially enhances atomic diffusivity. As a result, electromigration (EM) has become a critical reliability concern, causing severe microstructural degradation such as phase coarsening [1], the polarity [2,3] and the reverse polarity effects [4,5] and the formation of hillocks and voids [6,7].

Low-temperature soldering technology (<200 °C) has attracted considerable attention for large-chip assembly and consumer electronics due to its advantageous abilities to depress thermal stress, reduce warpage, improve assembly yield and conserve energy [8,9]. Currently, Sn-Bi-based lead-free solders, with melting temperatures ranging from 138 to 190 °C, have thus emerged as preferred candidates, offering lower processing temperatures than conventional Sn-3Ag-0.5Cu (SAC305, 217 °C). However, their lower melting points intensify thermal activation and accelerate atomic diffusion at typical service temperatures, thereby amplifying EM susceptibility [10,11].

Huang et al. [12,13] reported the EM-induced β-Sn/Bi phase separation in low-temperature Sn-57Bi-1Ag solder interconnects, observing continuous Bi layer segregation at the anode after 120 h of current stressing. Since the electrical resistivity of pure Bi (1.29 × 10^−6^ Ω·m) is significantly higher than those of eutectic Sn-58Bi solder (3.83 × 10^−7^ Ω·m) and pure Sn (1.10 × 10^−7^ Ω·m), this anodic Bi layer significantly increases the interconnect resistance, ultimately leading to EM failure. Therefore, suppressing these EM-induced kinetic processes is essential for enhancing the EM lifetime of Sn-Bi-based solder interconnects.

The Bi content fundamentally governs the microstructure and physical properties of Sn-Bi solders, including melting characteristics, electrical resistivity and atomic diffusivity [14,15,16]. It is anticipated that varying the Bi content should substantially influence the kinetics of EM-induced phase separation and anodic Bi layer growth, which in turn critically determines the EM-induced resistance evolution and overall EM lifetime. Despite its significance, systematic investigations on the role of Bi content in governing the EM failure mechanisms of those low-temperature Sn-Bi-based solder interconnects remain scarce.

In the present work, the EM-induced resistance variation and microstructural evolution in linear Cu/Sn-xBi-1Ag/Cu interconnects with various Bi contents were investigated. The dependence of EM lifetime on Bi content is quantitatively established, and the underlying physical mechanisms are discussed.

## 2. Experimental

Linear Cu/Sn-xBi-1Ag/Cu interconnects (x = 57, 47 and 40 wt.%) with a fixed spacing of 600 µm were fabricated by immersion soldering. Sequential electroplating was employed to deposit Ni and Cu films onto 10 mm × 10 mm × 10 mm Cu blocks. The Cu film served as the under bump metallization (UBM), while the Ni film served as a marker for tracking the Cu UBM consumption. A flux was applied to the electroplated Cu films, and the interconnect spacing was controlled using a 600 μm diameter stainless steel spacer.

Table 1 lists the Bi contents, soldering temperatures and adjusted EM test bath temperatures for three Cu/Sn-xBi-1Ag/Cu interconnects. The Sn-xBi-1Ag solders were melted in a crucible and maintained at temperatures of 190 °C (x = 57 wt.%), 210 °C (x = 47 wt.%) and 220 °C (x = 40 wt.%), respectively. Pairs of Cu blocks were immersion soldered for 10 s. The soldered assemblies were cross-sectioned into 600 μm × 600 μm specimens using electric discharge machining, then ground and polished to final dimensions of 300 μm × 300 μm.

EM testing was conducted in a silicone oil bath maintained at 90 °C for Sn-57Bi-1Ag, 92 °C for Sn-47Bi-1Ag and 94 °C for Sn-40Bi-1Ag. A current density of 7.5 × 10^3^ A/cm^2^ was applied, resulting in a steady-state interconnect temperature of 100 ± 1 °C due to Joule heating. Real-time resistance was monitored using the Kelvin four-probe method. The resistance-increase percentage *α*(*t*) over time *t* is defined as:

(1) $$\alpha (t)\;=\;\frac{R(t)-R_{0}}{R_{0}}\times 100\%$$

where *R*(*t*) is the time-dependent resistance, and *R*_0_ is the initial resistance.

Microstructural characterization was performed using a scanning electron microscope (SEM, IT800-SHL, JEOL, Tokyo, Japan) equipped with an energy dispersive X-ray (EDX) spectrometer (Oxford Instruments, Abingdon on Thames, UK). The thickness of the Bi layer was quantified via Photoshop image analysis (5 measurements per data point). The composition-dependent resistivity was simulated by ANSYS R2021a finite element analysis based on a two-phase microstructure model generated via a Cellular Automaton and Monte Carlo algorithm, in which phase-specific resistivities were assigned. The electric potential distribution under a 1 A direct current was simulated, from which the resistivity was calculated.

## 3. Results and Discussion

### 3.1. Microstructure of As-Soldered Sn-xBi-1Ag Solder Interconnects

**Figure 1:**
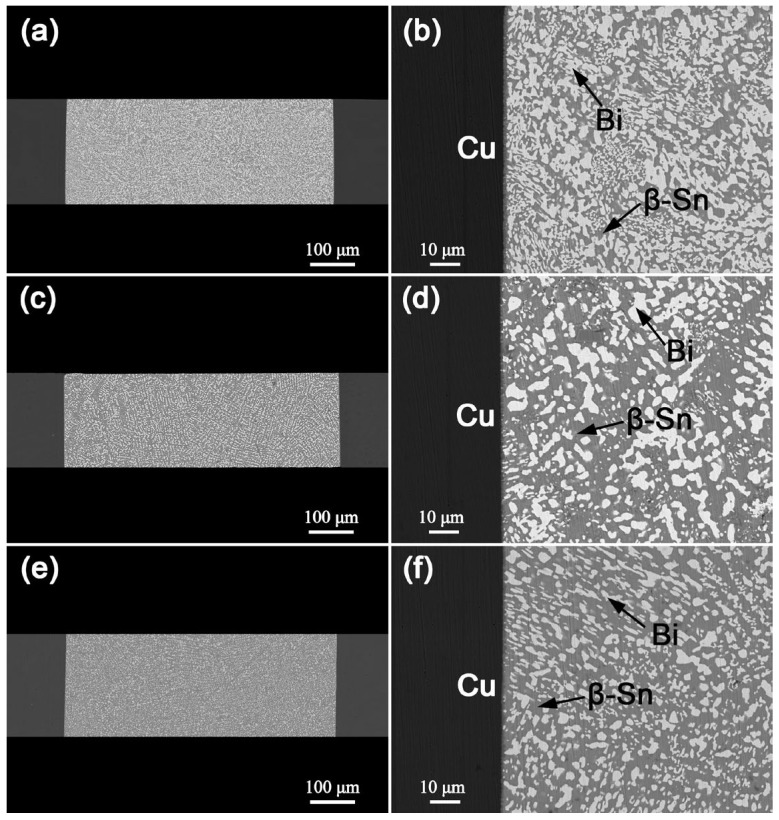
Microstructure of as-soldered Cu/Sn-xBi-1Ag/Cu interconnects: (**a**,**b**) x = 57, (**c**,**d**) x = 47 and (**e**,**f**) x = 40.

Figure 1 shows the cross-sectional SEM images of the as-soldered Cu/Sn-xBi-1Ag/Cu interconnects. All compositions exhibited void-free interfacial bonding and continuous Cu_6_Sn_5_ intermetallic compound (IMC) layers at the interfaces. The IMC thickness increased with decreasing Bi content, measuring 1.1 μm (x = 57), 1.5 μm (x = 47) and 1.7 μm (x = 40). This trend is attributed to the higher soldering temperatures required for solders with lower Bi content. Sn-57Bi-1Ag solder featured a near-eutectic microstructure, characterized by fine Bi phases uniformly dispersed in β-Sn matrix. In contrast, Sn-47Bi-1Ag and Sn-40Bi-1Ag exhibited a hypoeutectic microstructure dominated by primary β-Sn dendrites with discrete fine Bi phases.

### 3.2. Resistance Evolution Under Current Stressing

**Figure 2:**
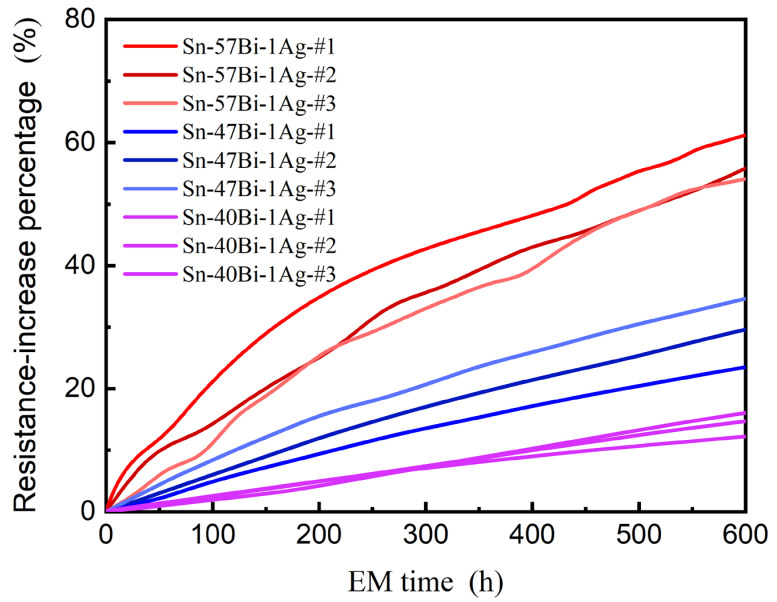
Resistance evolution of Cu/Sn-xBi-1Ag/Cu interconnects under a current density of 7.5 × 10^3^ A/cm^2^ at 100 °C. Lines #1, #2, and #3 correspond to data obtained from three separate interconnects tested for each composition.

**Figure 3:**
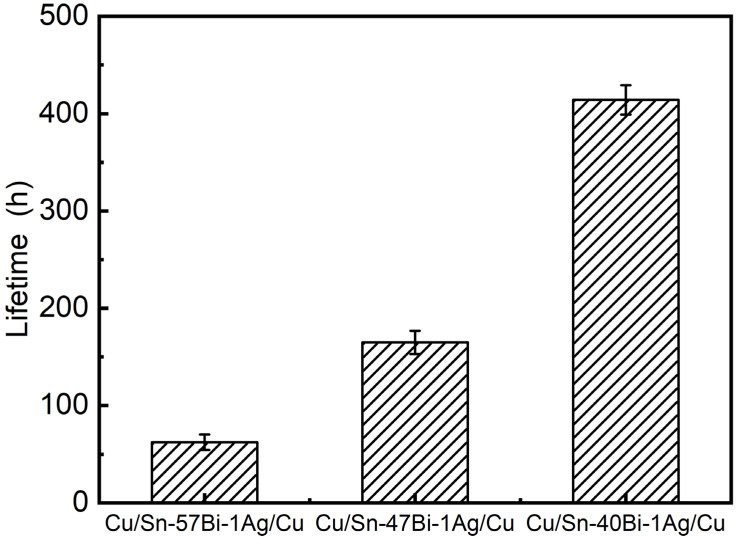
Lifetimes of Cu/Sn-xBi-1Ag/Cu interconnects at the failure criterion of 10% resistance increase.

Figure 2 shows the resistance evolution of the Cu/Sn-xBi-1Ag/Cu interconnects under a current density of 7.5 × 10^3^ A/cm^2^ at 100 °C. All samples exhibited monotonic resistance increases, with kinetics strongly modulated by the Bi content. Sn-57Bi-1Ag solder interconnects demonstrated the most rapid resistance growth increase, whereas reducing the Bi content progressively suppressed the resistance increases in Sn-47Bi-1Ag and Sn-40Bi-1Ag solder interconnects. This correlation is further quantified in Figure 3, which plots the EM lifetimes (defined as the time to reach a 10% resistance increase) for different compositions. The measured lifetime increased from 62.3 h (x = 57) to 164.9 h (x = 47) and 414.1 h (x = 40), representing 2.6-fold and 6.6-fold improvements, respectively. These enhancements originated from the suppression of EM-induced microstructural degradation.

The EM lifetimes reported in this study were obtained under accelerated test conditions. For a extrapolation to typical service conditions (e.g., 80 °C, 1.0 × 10^3^ A/cm^2^), the empirical Black’s equation is often used $t_{f}=Aj^{-n}\exp(E_{a}/kT)$, where *t_f_* is the EM lifetime, *A* is the pre-factor constant of material, *j* is the current density, *n* is the current density exponent, *E_a_* is the activation energy, *k* is the Boltzmann constant and *T* is the EM temperature). Using the values from literature [11] for Sn-Bi-based solder interconnects (*n* = 1, *E_a_* = 0.81 eV), the acceleration factor from the test condition to the service condition is calculated to be 31.3. Based on this, the EM lifetimes for Sn-57Bi-1Ag, Sn-47Bi-1Ag and Sn-40Bi-1Ag interconnects are approximately 1951 h, 5163 h and 12,965 h, respectively. It is important to note that the Black’s equation was originally established for void formation in homogeneous metallic lines, which is distinct from the dominant β-Sn/Bi phase separation and anodic Bi layer growth observed in Sn-Bi-based solder interconnects. Nevertheless, as a widely adopted empirical correlation, it offers a useful first-order estimate for comparative lifetime prediction. In this empirical framework, the extrapolation consistently confirms that reducing the Bi content substantially extends the EM lifetime.

### 3.3. Current-Stressing-Induced Microstructural Evolution

**Figure 4:**
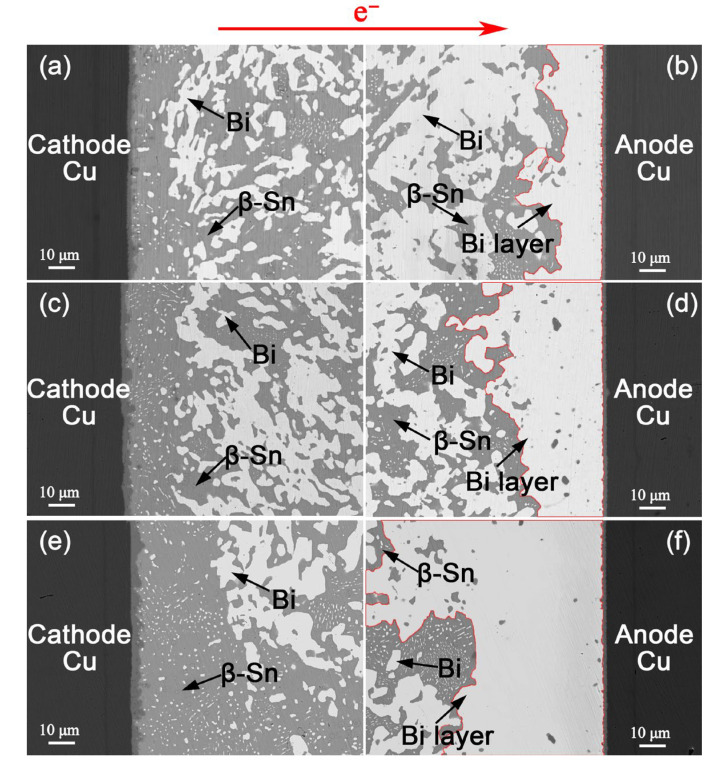
Microstructure of Cu/Sn-57Bi-1Ag/Cu interconnects under a current density of 7.5 × 10^3^ A/cm^2^ at 100 °C for: (**a**,**b**) 200 h, (**c**,**d**) 400 h and (**e**,**f**) 600 h. The red lines indicate the anodic Bi layer.

Figure 4 shows the microstructural evolution of the Cu/Sn-57Bi-1Ag/Cu interconnects under a current density of 7.5 × 10^3^ A/cm^2^ at 100 °C. EM drove pronounced migration of Bi atoms towards the anode, resulting in progressive β-Sn/Bi phase separation and the formation of a continuous Bi layer at the anode. With extended current stressing time, the segregated anodic Bi layer thickened linearly, and the residual Bi phases within the solder matrix coarsened. After EM for 600 h, the anodic Bi layer reached 69.4 μm in thickness and the coarsened Bi particles grew to 29.3 μm in diameter. Although the coarsening of Bi particles is known to slightly reduce interconnect resistance [13], a monotonic overall increase was still observed during EM (Figure 2). This net resistance increase confirms that the formation of anodic Bi layer is the dominant factor, overriding the competing effect of Bi particle coarsening.

**Figure 5:**
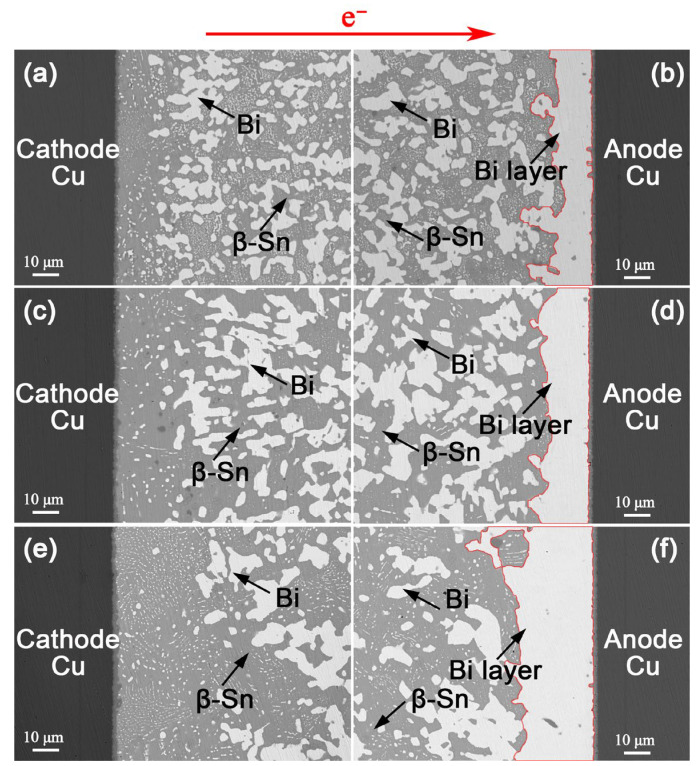
Microstructure of Cu/Sn-47Bi-1Ag/Cu interconnects under a current density of 7.5 × 10^3^ A/cm^2^ at 100 °C for: (**a**,**b**) 200 h, (**c**,**d**) 400 h and (**e**,**f**) 600 h. The red lines indicate the anodic Bi layer.

**Figure 6:**
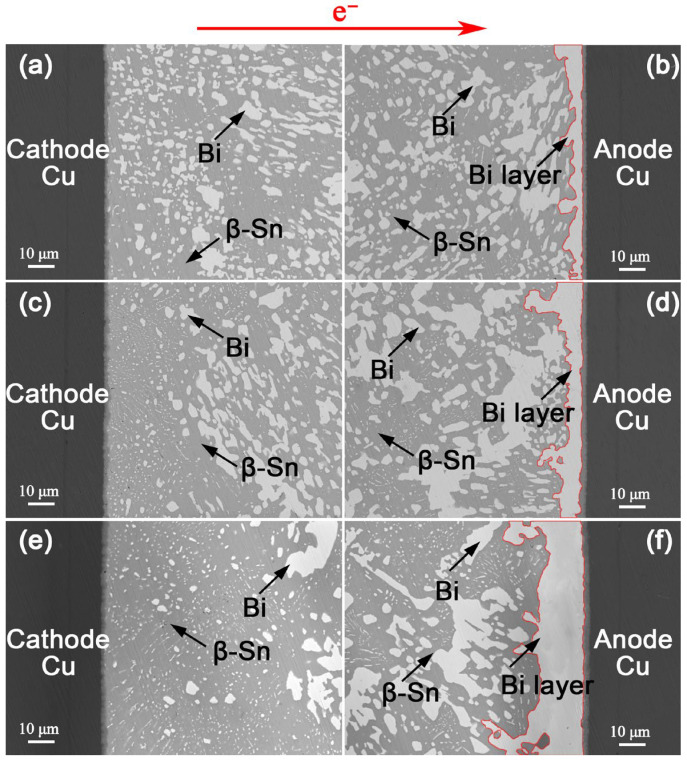
Microstructure of Cu/Sn-40Bi-1Ag/Cu interconnects under a current density of 7.5 × 10^3^ A/cm^2^ at 100 °C for: (**a**,**b**) 200 h, (**c**,**d**) 400 h and (**e**,**f**) 600 h. The red lines indicate the anodic Bi layer.

Figure 5 and Figure 6 present analogous microstructural evolutions in the Sn-47Bi-1Ag and Sn-40Bi-1Ag solder interconnects, respectively. After EM for 600 h, the thicknesses of the continuous anodic Bi layers were 31.0 μm and 22.5 μm, representing reductions of 55.3% and 67.6%, respectively, compared to the Sn-57Bi-1Ag solder interconnect.

**Figure 7:**
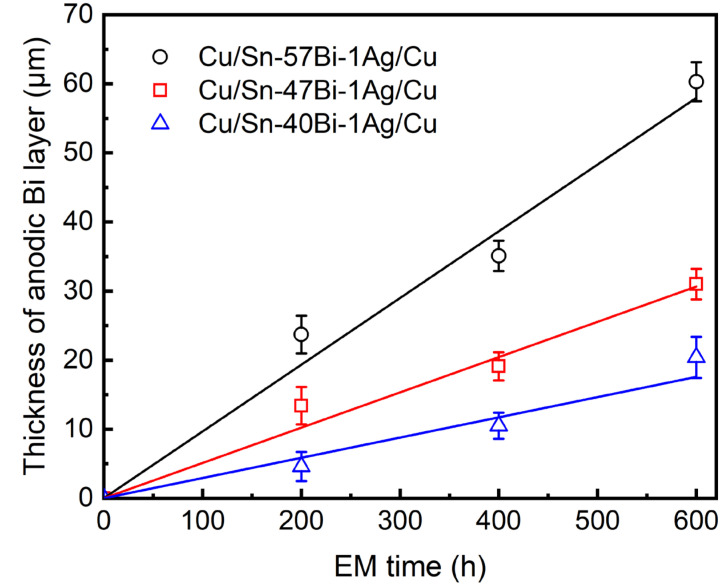
Growth kinetics of anodic Bi layer in Cu/Sn-xBi-1Ag/Cu interconnects under a current density of 7.5 × 10^3^ A/cm^2^ at 100 °C.

Figure 7 shows the growth kinetics of the anodic Bi layers in Sn-xBi-1Ag solder interconnects, revealing a strong linear correlation between layer thickness and EM time for all compositions (R^2^ > 0.9). Crucially, reducing the Bi content significantly suppressed the growth rate from 0.114 μm/h (x = 57) to 0.051 μm/h (x = 47) and 0.035 μm/h (x = 40). This confirms that Bi content reduction is the dominant mechanism for suppressing anodic Bi layer segregation and extending EM lifetime.

### 3.4. Theoretical Analysis of Bi Content on EM Failure Mechanism

Since the product of diffusivity (*D*) and effective charge number (Z*) of Bi atoms (DZ* = 4.72 × 10^−10^ cm^2^/s [17]) is one order of magnitude higher than that of Sn atoms (DZ* = 1.57 × 10^−11^ cm^2^/s [2,18]), Bi atoms are the primary diffusing species during EM. The EM-induced Bi atomic flux (*J_EM_*), which governs the growth of anodic Bi layer, is expressed as:

(2) $$J_{EM}\;=\frac{CD}{kT}e\rho JZ^{*}$$

where *C* is the atomic concentration of Bi (number density), *D* is the diffusion coefficient, *k* is the Boltzmann constant, *T* is the absolute temperature, *e* is the electronic charge, *ρ* is the electrical resistivity, *j* is the current density and Z* is the effective charge number. It is crucial to note that *C*, *ρ* and *D* are strongly composition-dependent, collectively governing the EM flux dynamics.

**Figure 8:**
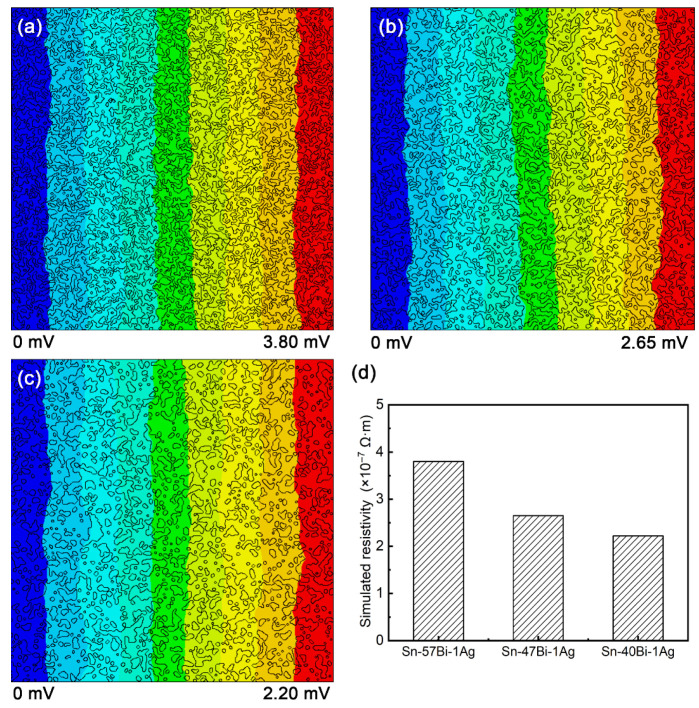
Simulated potential distributions in Sn-xBi-1Ag solders under a 1 A current: (**a**) Sn-57Bi-1Ag, (**b**) Sn-47Bi-1Ag and (**c**) Sn-40Bi-1Ag, and (**d**) simulated resistivities of Sn-xBi-1Ag solders.

Naturally, a reduction in Bi content directly lowers the atomic concentration (*C*) of mobile Bi atoms. Meanwhile, the electrical resistivities (*ρ*) of Sn-xBi solders were calculated using the fundamental relationship $\rho \;={\;R}_{x}\frac{S}{L}$, where *R_x_* is the electrical resistance, *S* is the cross-sectional area and *L* is the length of solder. The key parameter *R_x_* was derived from simulated electric potential distributions in Sn-xBi solders under a 1 A current (Figure 8a–c), with neglecting the minor influence of Ag. The electrical resistivity progressively declines from 3.90 × 10^−7^ Ω·m for Sn-57Bi (closely matching the reported value of 3.83 × 10^−7^ Ω·m for eutectic Sn-58Bi [19]) to 2.58 × 10^−7^ Ω·m for Sn-47Bi and 2.20 × 10^−7^ Ω·m for Sn-40Bi, as shown in Figure 8d. This reduction stems from the increasing volumetric dominance of low-resistivity Sn (1.10 × 10^−7^ Ω·m) over high-resistivity Bi (1.29 × 10^−6^ Ω·m).

The influence of Bi content on diffusivity is governed by two main microstructural diffusion pathways: high-activation-energy lattice diffusion and low-activation-energy phase boundary diffusion. The corresponding coefficients (*D_l_* and *D_pb_*) are expressed as:

(3) $$D_{L}\;={\;D}_{0}exp(-\frac{Q_{L}}{kT})$$

(4) $$D_{pb}={\;D}_{0}exp(-\frac{Q_{gb}}{kT})$$

where *D*_0_ is the diffusion constant, *k* is the Boltzmann constant, *T* is the absolute temperature, *Q_l_* is the activation energy for lattice diffusion (*Q_l_* = 99.72 kJ/mol for Bi atoms [20]) and *Q_pb_* is the activation energy for phase boundary diffusion. The phase boundary diffusion activation energy (*Q_pb_*) is commonly expressed as a function of the lattice diffusion activation energy (*Q_l_*):

(5) $$Q_{pb}\;=\;\alpha \;\times {\;Q}_{L}$$

where *α* is a dimensionless factor typically ranging from 0.5 to 0.7 for metallic systems; a value of *α* = 0.6 is adopted in this case. The ratio *δ*, defined as *D_l_*/*D_pb_*, is expressed as:

(6) $$\delta \;=\;\frac{D_{L}}{D_{pb}}\;=\;exp(\frac{Q_{L}-Q_{pb}}{kT})\;=\;exp(\frac{{(1-\alpha )Q}_{L}}{kT})$$

The calculated result (*δ* = 1.1 × 10^−7^) confirms that *D_l_* ≪ *D_pb_*, emphasizing the critical role of β-Sn/Bi phase boundaries as fast diffusion channels.

**Figure 9:**
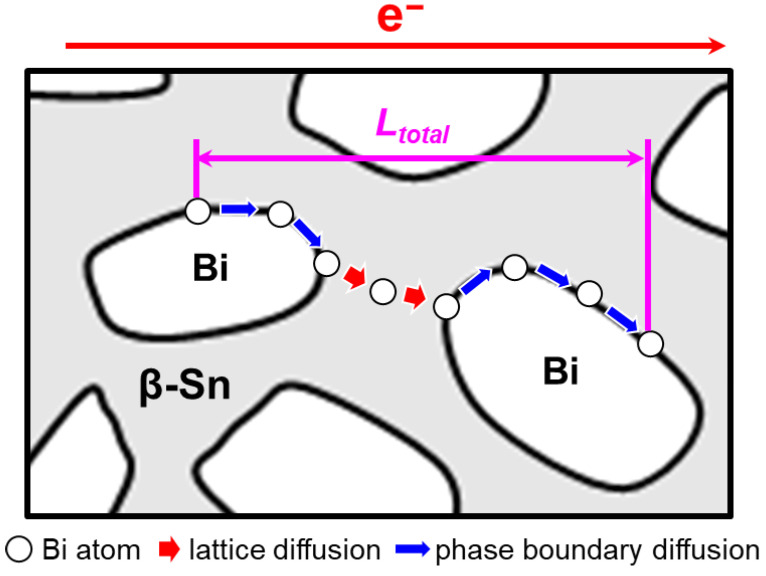
Schematics of Bi atomic diffusion process under current stressing.

The discontinuous nature of the β-Sn/Bi phase boundaries drives the periodic shuttling of Bi atoms between the fast diffusion channels along the boundaries and the slower β-Sn matrix lattice (Figure 9). According to the Einstein-Smoluchowski equation, the diffusion coefficient (*D*) is defined by the mean square diffusion distance (<*L*^2^>) of Bi atoms for the diffusion time (*t*):

(7) $$D\;=\;\frac{<L^{2}>}{2\times dim\times t}$$

where *dim* is the space dimension. Therefore, the total diffusion time can be modeled as the sum of the diffusion times from each segment:

(8) $$\frac{L_{\mathrm{total}}^{2}}{D_{\mathrm{eff}}}\;=\;\frac{L_{\mathrm{Sn}}^{2}}{D_{L}}\;+\;\frac{L_{pb}^{2}}{D_{pb}}$$

where *L_total_* is the total diffusion distance, *D_eff_* is the effective diffusion coefficient, *L_Sn_* is the lattice diffusion distance and *L_pb_* is the phase boundary diffusion distance. Therefore, the effective diffusion coefficient is expressed as:

(9) $$D_{\mathrm{eff}}\;=\frac{L_{\mathrm{total}}^{2}}{\frac{L_{\mathrm{Sn}}^{2}}{D_{L}}\;+\;\frac{L_{pb}^{2}}{D_{pb}}}$$

The Bi volume fraction (*V_V_*) is defined as:

(10) $$V_{V}\;=\;\frac{V_{\mathrm{Bi}}}{V_{\mathrm{total}}}$$

where *V_Bi_* is the volume of Bi and *V_total_* is the total volume of solder. The specific surface area of Bi (*S_V_*) is expressed as:

(11) $$S_{V}\;=\;\frac{S_{\mathrm{total}}}{V_{\mathrm{total}}}\;=\;\frac{N\overset{-}{S}}{V_{\mathrm{total}}}\;=\;\frac{N\overset{-}{S}V_{V}}{V_{\mathrm{Bi}}}\;=\;\frac{N\overset{-}{S}V_{V}}{N\overset{-}{V}}\;=\;\frac{\overset{-}{S}}{\overset{-}{V}}V_{V}$$

where *S_total_* is the total surface area, *N* is the number of Bi phases, $\bar{s}$ is the average surface area and $\bar{v}$ is the average volume of Bi phases. A proportionality coefficient *β* is introduced to relate the ratio of average surface area to volume ($\frac{\bar{s}}{\bar{v}}$) to the inverse of the average Bi phase size ($\frac{1}{\overset{-}{d}}$):

(12) $$\frac{\overset{-}{S}}{\overset{-}{v}}\;=\;\frac{\beta }{\overset{-}{d}}$$

where *β* is a geometrical factor that depends on the shape of Bi phases and $\bar{d}$ is the average size of Bi phases. The relationship between the three-dimensional specific surface area (*S_V_*) and the two-dimensional crystal boundary fraction (*f*) is given by the fundamental stereological relationship [21]:

(13) $$S_{V}\;=\;\frac{4}{\pi }f$$

Additionally, a scaling factor *m* is introduced to relate the phase boundary diffusion distance (*L_pb_*) to the average Bi phase size ($\bar{d}$):

(14) $$L_{pb}\;=\;m\overset{-}{d}$$

where *m* is the tortuosity of diffusion pathway along the phase boundaries. Solving the system of equations yields:

(15) $$L_{pb}\;=\;\frac{m\pi \beta V_{V}}{4f}$$

Substituting Equation (15) into Equation (9) gives the final expression for the effective diffusion coefficient:

(16) $$D_{\mathrm{eff}}\;=\;\frac{L_{\mathrm{total}}^{2}}{\frac{L_{\mathrm{Sn}}^{2}}{D_{L}}\;+\;{\left(\frac{m\pi \beta V_{V}}{4f}\right)}^{2}\;\times \;\frac{1}{D_{pb}}}$$

**Figure 10:**
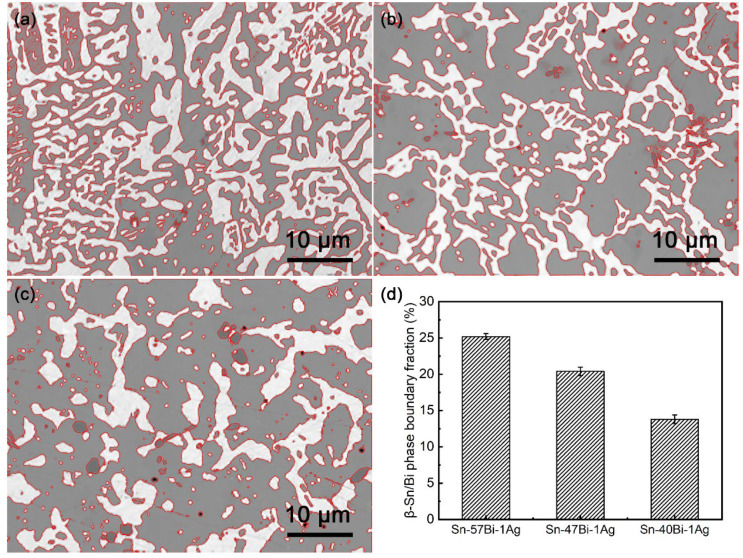
Image-processed microstructure of: (**a**) Sn-57Bi-1Ag, (**b**) Sn-47Bi-1Ag and (**c**) Sn-40Bi-1Ag, and (**d**) calculated β-Sn/Bi phase boundary fractions.

A reduction in Bi content enhances the dominance of β-Sn phases, which increases the spacing between Bi phases and thereby extends the lattice diffusion distance (*L_Sn_*). Quantitative microstructural analysis (Figure 10) reveals that reducing the Bi content (*V_V_*) leads to a decrease in phase boundary fraction (*f*), indicating a reduced density of fast diffusion pathways. The normalized results in Table 2 (with Sn-57Bi-1Ag as reference) shows a slight but steady increase in $({\frac{V_{V}}{f})}^{2}\;$ as the Bi content decreases. Although the increase in $({\frac{V_{V}}{f})}^{2}$ is not substantial, it contributes to an overall increase in phase boundary diffusion. As a result, the increases in *L_Sn_* and $({\frac{V_{V}}{f})}^{2}$ enhance the $\frac{L_{Sn}^{2}}{D_{L}}$ and ${\left(\frac{m\pi \beta V_{V}}{4f}\right)}^{2}\;\times \;\frac{1}{D_{pb}}$, respectively, ultimately suppressing the effective diffusion coefficient (*D_eff_*) of Bi atoms.

Given that *D_l_* ≪  *D_pb_*, the phase boundary diffusion can be neglected, enabling a quantitative approximation of the effective diffusion coefficient (*D_eff_*):

(17) $$D_{\mathrm{eff}}\;\approx \;\frac{L_{\mathrm{total}}^{2}}{L_{\mathrm{Sn}}^{2}}D_{L}$$

In this simplified model, the lattice diffusion distance (*L_Sn_*) scales with the characteristic spacing between Bi phases, which depends on the volume fraction of β-Sn matrix. This geometric consideration leads to the relation $\frac{L_{\mathrm{total}}}{L_{\mathrm{Sn}}}\;=\;\frac{1}{1-V_{V}}$, resulting in:

(18) $$D_{\mathrm{eff}}\;\approx \;{\left(\frac{1}{1-V_{V}}\right)}^{2}D_{L}$$

**Figure 11:**
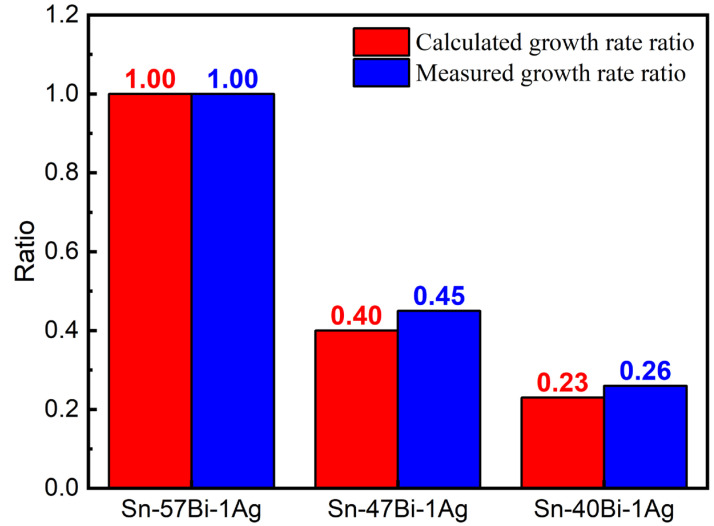
Calculated and measured Bi layer growth rate ratios normalized to Sn-57Bi-1Ag.

According to Equation (18), the effective diffusion coefficients of Bi atoms in Sn-57Bi-1Ag, Sn-47Bi-1Ag and Sn-40Bi-1Ag solder joints are calculated to be 3.95-fold, 2.76-fold and 2.24-fold the lattice diffusion coefficient, respectively. Combined with the reductions in atomic concentration (*C*) and electrical resistivity (*ρ*), these three factors collectively suppress the EM-induced Bi atomic flux (*J_EM_*). Since the growth rate of anodic Bi layer is proportional to *J_EM_*, the normalized *J_EM_* ratios (with Sn-57Bi-1Ag as reference) directly represent the theoretically calculated growth rate ratios (1:0.40:0.23). Figure 11 shows close agreement between these calculated ratios and the measured anodic Bi layer growth rate ratios (1:0.45:0.26), confirming that the suppression of atomic flux is the governing mechanism for EM failure retardation.

As a result, the enhanced EM reliability stems from Bi content reduction through three different mechanisms: (i) a decrease in the atomic concentration of mobile Bi atoms; (ii) a reduction in electrical resistivity that weakens the electron wind force; and (iii) an increase in lattice diffusion distance that lowers the effective diffusion coefficient (D_eff_). These mechanisms collectively suppress Bi migration, thereby inhibiting phase separation and delaying anodic layer growth. Consequently, the EM lifetime increases substantially from 62.3 h for Sn-57Bi-1Ag to 164.9 h for Sn-47Bi-1Ag and 414.1 h for Sn-40Bi-1Ag, representing 2.6-fold and 6.6-fold improvements, respectively.

## 4. Conclusions

1. The electromigration behavior of Sn-xBi-Ag solder interconnects is dominated by Bi atom migration, driven by its intrinsically higher DZ* value (4.72 × 10^−10^ cm^2^/s) compared to that of Sn atoms (1.57 × 10^−11^ cm^2^/s), resulting in dynamic β-Sn/Bi phase separation and linear growth of an anodic Bi layer.
2. Reducing the Bi content suppresses the EM-induced Bi atomic flux (*J_EM_*) through three principal mechanisms: (i) a decrease in the atomic concentration of mobile Bi atoms; (ii) a reduction in electrical resistivity that weakens the electron wind force; and (iii) an increase in lattice diffusion distance that lowers the effective diffusion coefficient (*D_eff_*).
3. The close agreement between the calculated (1:0.40:0.23) and measured (1:0.45:0.26) anodic Bi layer growth rate ratios demonstrates that EM-induced atomic flux is the dominant factor controlling failure in Sn-Bi-Ag-based solder interconnects.
4. Reducing the Bi content from 57% to 47% and 40% suppresses the anodic Bi layer growth rate from 0.114 μm/h to 0.051 μm/h and 0.035 μm/h, respectively, thereby significantly extending the EM lifetime from 62.3 h to 164.9 h and 414.1 h, corresponding to 2.6-fold and 6.6-fold improvements.

## Figures and Tables

**Table 1:** Bi content, soldering temperature and EM test bath temperature for Cu/Sn-xBi-1Ag/Cu interconnects.

Bi Content (wt.%)	Soldering Temperature (°C)	EM Test Bath Temperature (°C)
57	190	90
47	210	92
40	220	94

**Table 2:** Ratios normalized to Sn-57Bi-1Ag.

	Sn-57Bi-1Ag	Sn-47Bi-1Ag	Sn-40Bi-1Ag
$({\frac{V_{V}}{f})}^{2}$	1.00	1.08	1.31

## Data Availability

The data presented in this study are available on request from the corresponding author. The data are not publicly available due to privacy or ethical restrictions.

## References

[1] Huang M.L., Zhao J.F., Zhang Z.J., Zhao N. (2016). Dominant effect of high anisotropy in β-Sn grain on electromigration-induced failure mechanism in Sn-3.0Ag-0.5Cu interconnect. J. Alloys Compd..

[2] Huang M.L., Zhao J.F., Zhang Z.J., Zhao N. (2015). Role of diffusion anisotropy in β-Sn in microstructural evolution of Sn-3.0Ag-0.5Cu flip chip bumps undergoing electromigration. Acta Mater..

[3] Fu Z.W., Wei Q.R., Guo X.T., Fu X., Wang J., Yang C., Guo H.X., Yang J.Y. (2023). Influence of temperature and current stressing on Cu-Sn intermetallic compound growth characteristics of lead-free microbump. Adv. Theory Simul..

[4] Huang M.L., Zhang Z.J., Zhao N., Zhou Q. (2013). A synchrotron radiation real-time in situ imaging study on the reverse polarity effect in Cu/Sn-9Zn/Cu interconnect during liquid-solid electromigration. Scr. Mater..

[5] Zhang X.F., Guo J.D., Shang J.K. (2007). Abnormal polarity effect of electromigration on intermetallic compound formation in Sn-9Zn solder joint. Scr. Mater..

[6] Wang S.B., Liu P., Cong S., Guo W.Q., Zhang W.W. (2024). The evolution of micro-voids in Sn37Pb solder joints under electromechanical coupling loading. J. Electron. Mater..

[7] Li C., Yuan H.Y., Ma Z.L., Cheng X.W. (2024). Effect of βSn grain orientations on the electromigration-induced evolution of voids in SAC305 BGA solder joints. Mater. Charact..

[8] Wang F.J., Chen H., Huang Y., Liu L.T., Zhang Z.J. (2019). Recent progress on the development of Sn-based low-temperature Pb-free solders. J. Mater. Sci. Mater. Electron..

[9] Kang H., Rajendran S.H., Jung J.P. (2021). Low melting temperature Sn-Bi solder: Effect of alloying and nanoparticle addition on the microstructural, thermal, interfacial bonding, and mechanical characteristics. Metals.

[10] Huang J.Q., Wang X.D., Chen J.Y., Wei W.C., Liu F.M., Qin B.H., Wang H.Y., Zhang Y.P. (2022). Growth mechanisms of intermetallic compounds and Bi-rich layer in ball grid array structure Cu/Sn-58Bi/Cu solder joints during solid-solid and liquid-solid electromigration. J. Mater. Sci. Mater. Electron..

[11] Flores J., Panta S., Hadian F., Cotts E. (2024). Changes in the microstructure and electrical resistance SnBi-based solder joints during current stressing. J. Electron. Mater..

[12] Li L., Wang S.B., Chen J.L., Huang M.L. Electromigration-induced failure behavior of low-temperature Cu/Sn-57Bi-1Ag/Cu solder joints. Proceedings of the 2023 24th International Conference on Electronic Packaging Technology (ICEPT).

[13] Chen J.L., Wang S.B., Ren J., Huang M.L. (2024). Effect of annealing treatment on electromigration resistance of low-temperature Sn-57Bi-1Ag solder interconnect. J. Electron. Mater..

[14] Du C.C., Ye D., Wu M.F., Lai Z.M. (2015). Microstructure and mechanical properties of Sn-xBi solder alloy. J. Mater. Sci. Mater. Electron..

[15] Takao H., Yamada A., Hasegawa H., Matsui M. (2002). Mechanical properties and solder joint reliability of low-melting Sn-Bi-Cu lead free solder alloy. J. Jpn. Inst. Electron. Packag..

[16] Ribas M., Chegudi S., Kumar A., Pandher R., Raut R., Mukherjee S., Sarkar S., Singh B. Development of low-temperature drop shock resistant solder alloys for handheld devices. Proceedings of the 15th Electronics Packaging Technology Conference 2013.

[17] Chen L.T., Chen C.M. (2006). Electromigration study in the eutectic SnBi solder joint on the Ni/Au metallization. J. Mater. Res..

[18] Ho P.S., Kwok T. (1989). Electromigration in metals. Rep. Prog. Phys..

[19] Ren J., Huang F.F., Li A.J., Huang M.L. (2025). Electromigration-induced failure mode of low-temperature Sn-49Bi-1Ag hybrid BGA joint in flip chip package. J. Mater. Res. Technol.-JMRT.

[20] Delhaise A.M., Perovic D. (2018). Study of solid-state diffusion of Bi in polycrystalline Sn using electron probe microanalysis. J. Electron. Mater..

[21] Baddeley A.J., Gundersen H.J.G., Cruz-Orive L.M. (1986). Estimation of surface area from vertical sections. J. Microsc..

